# Aortic Aneurysm

**Published:** 1890-02-01

**Authors:** 


### AORTIC ANEURYSM.

Dr. Douglas Powell, physician to the Middlesex Hospital, has some remarks (*Lancet*, Jan. 4) on the diagnosis and treatment of aneurysm of the aorta. Pathologically, aneurysms are divided into those in which the whole circumference of the vessel is dilated, and those in which the vessel yields at some one point from which projects a sac. The one is known as the *fusiform*, or globular aneurysm, the other as the *sacculated* aneurysm. But, clinically, the phenomena characteristic of aneurysm are scarcely ever observed except in association with the sacculated form, the fusiform aneurysm being merged as regards prognosis in the class of heart diseases. The fusiform dilatations are not to be treated by rest, starvation diet, iodide of potassium, with a view to bringing about coagular depositions. Their treatment is that appropriate to cardiac disease, for they do not cause death by rupture or compression of vital parts, but rather through heart failure, anguina, and syncope. The question of diagnosis turns on whether there are symptoms of pressure or not, which do not generally arise from a general dilatation of the vessel affected.

